# RadioLabeling of the Mutant form of Anti-PlGF Nanobody by ^99m^Tc-Tricarbonyl

**DOI:** 10.5812/ijpr-144901

**Published:** 2024-06-02

**Authors:** Tahereh Rezazadeh, Akram Sadat Tabatabaee Bafroee, Soraya Shahhosseini, Safura Jokar, Abolfazl Hasanramezani, Roghaye Arezumand

**Affiliations:** 1 Department of Advanced Sciences and Technologies, School of Medicine, North Khorasan University of Medical Sciences, Bojnurd, Iran; 2 Department of Biology, East Tehran Branch, Islamic Azad University, Tehran, Iran; 3 Department of Pharmaceutical Chemistry and Radiopharmacy, Protein Technology Research Center, Shahid Beheshti University of Medical Sciences, Tehran, Iran; 4 Department of Nuclear Pharmacy, Faculty of Pharmacy, Tehran University of Medical Sciences, Tehran, Iran

**Keywords:** PlGF, Radiolabeled Nanobody, ^99m^Tc-tricarbonyl

## Abstract

**Background:**

Molecular imaging is a highly effective method for diagnosing cancer and evaluating treatment. A molecular tracer often consists of two segments: A targeting segment, which can be antibodies, antibody fragments, or VHH (nanobody), and a detection segment, such as radioisotopes. The small size of VHH allows for excellent tissue penetration and fast clearance, resulting in minimal nonspecific background, which makes them appealing for use as imaging agents. ^99m^-technetium (^99m^Tc), one of the well-known radioisotopes, is particularly useful in routine clinical imaging.

**Objectives:**

This study aims to construct ^99m^Tc-anti-placenta growth factor (PlGF) nanobody and assess its radiochemical purity (RCP).

**Methods:**

The mutant form of anti-PlGF nanobody was expressed in *E. coli TG1* and purified using Ni-NTA column affinity chromatography. The purified nanobodies were confirmed by SDS-PAGE and western blotting. A ^99m^Tc-tricarbonyl solution was added to phosphate-buffered saline (PBS) containing the mutant nanobody for labeling, and the mixture was purified using a PD-10 column.

**Results:**

The RCP of ^99m^Tc-tricarbonyl is > 98%. After the addition of radioisotopes to the mixture of nanobodies, purity reached 70% in 2 hours and remained constant during incubation. After purifying the labeled nanobody, activity was measured, and the highest amount of labeled nanobodies was collected in the second part. The stability of the labeled nanobody in PBS and in competition with histidine for 4 hours was checked by thin-layer chromatography (TLC).

**Conclusions:**

The findings of this study reveal that the RCP of the labeled nanobody was above 95% after 4 hours, indicating the labeled antibody's stability. These results are promising and could be utilized in future in vitro and in vivo studies.

## 1. Background

The early detection of cancer is crucial for its treatment, and regular cancer screenings are key in effectively monitoring a patient's clinical status. Thus, diagnostic and therapeutic imaging methods are indispensable. Techniques such as magnetic resonance imaging (MRI), computed tomography (CT), PET, and SPECT provide comprehensive, non-invasive scans that help characterize tumors in terms of their cellular conditions.

Among the available imaging methods, molecular imaging stands out by allowing the investigation of biological processes at molecular and cellular levels within living organisms. Molecular tracers, which consist of a targeting segment to guide the tracer and a signaling segment for detection, are used to identify biomarkers expressed in pathophysiological processes (1). Various biomolecules like antibodies and their derivatives are utilized for targeting, while substances such as fluorescence, enzymes, and radioisotopes serve as signaling agents.

PET and SPECT, which are molecular imaging modalities, utilize radioisotopes; PET employs isotopes that emit positrons, such as ^11^C, ^68^Ga, ^18^F, ^64^Cu, ^99m^Tc, or ^89^Zr, while SPECT uses isotopes like ^99m^Tc or ^111^In that emit gamma rays (2). 

Technetium-99m (^99m^Tc), extensively used in routine clinical SPECT imaging, is favored due to its optimal physical radiation characteristics, which are ideal for radiolabeling and imaging studies. With a half-life of 6 hours and a primary emission of 140 keV, ^99m^Tc can be readily produced by milking ^99^Mo/^99m^Tc generators (1). It is not only cost-effective but also well-understood in its chemical properties as a transition metal (3). The ^99m^Tc-tricarbonyl core, known as ^99m^Tc (I)-tricarbonyl, is small and spherical, capable of forming stable octahedral complexes when coordinated with biomolecules that possess suitable functional groups. It efficiently forms stable coordinate covalent bonds with heterocyclic rings containing nitrogen atoms such as imidazoles, pyridines, and pyrazoles. Notably, the imidazole ring of the histidine molecule can rapidly form a stable octahedral complex with technetium tricarbonyl, which remains highly stable due to the protection of technetium from reoxidation or further ligand attacks (3).

Nanobodies, which are the smallest fragments of natural antibodies, fulfill many criteria necessary for effective molecular tracers and can be labeled with fluorescent, bioluminescent, and radioisotopes. Due to their small size, high solubility, specificity, sensitivity, and excellent tissue permeability (4), nanobodies can quickly reach their targets within hours after injection, making them highly effective for tumor diagnosis and identification (5, 6). They offer advantages over complete antibodies, which have a molecular weight of 150 kDa and a longer half-life, thus providing greater practicality (7).

One significant pathological feature of cancer is the overexpression of angiogenesis. Targeting angiogenesis could thus be a potent strategy for cancer imaging. The placenta growth factor (PlGF), a pleiotropic cytokine from the VEGF family, selectively binds to VEGFR-1 and enhances VEGF's angiogenic effects. Targeting PlGF can potentially inhibit the growth and metastasis of tumor cells (8); hence, developing a high-affinity binder against PlGF could be beneficial for angiogenesis targeting. In this research, we utilized a mutant form of an anti-PlGF nanobody from a previous study, which demonstrated an enhanced affinity of approximately 5.8 X 10-4 nM (9).

The mutant nanobody was radiolabeled with technetium tricarbonyl for the first time, and its parameters were assessed.

## 2. Objectives

This study aimed to express the mutant form of the anti-PlGF nanobody in *E. coli TG1* and purify it using Ni-NTA affinity chromatography. We also aim to determine the radiochemical purity (RCP) and stability of the labeled nanobodies in phosphate buffer and when competing with histidine.

## 3. Methods

### 3.1. Expression and Purification of Mutant Nanobody Against PlGF

The vector used in this study was pHEN6c (provided by Professor Serge Muyldermans, VUB, Belgium), specifically pHEN6c-anti PlGF Nb, obtained from a previous study (9). This vector contains the lac operon, and IPTG (isopropylβ-D-1-thiogalactopyranoside) was used to induce protein expression. Initially, the vector was transformed into the cloning host (*E. coli* DH5α, Pasteur Institute, Iran) using the chemical method. After confirmation, pHEN6c-anti PlGF Nb was transformed into the expression host (*E. coli TG1* bacteria) (Pasteur Institute, Iran). A concentration of 1 mM IPTG (Cinaclon, Iran) was used to induce protein expression.

The periplasmic extract was then harvested using TES (10) (Tris, EDTA, 0.5M Sucrose) buffer, and purification was performed using a Ni-NTA affinity chromatography column (QIAGEN, Germany). The molecular weight of the purified nanobody was determined by SDS-PAGE (Bio-Rad, USA) (11), and the identity of the target protein was confirmed via western blotting (Bio-Rad, USA). To prepare the protein for the labeling process and to remove imidazole, the protein solution was dialyzed using a dialysis bag with a 3.5 kDa cut-off (Serva, Germany). To enhance the efficiency of the labeling process and the binding of the radioisotope to the nanobody, the volume of the protein solution was reduced using a freeze-dryer (Operon, South Korea) until it reached the required concentration.

### 3.2. Preparation and Confirmation of 99mTc-Tricarbonyl Complex

The ^99m^Tc-tricarbonyl complex, [^99m^Tc(H_2_O)_3_(CO)_3_]^+^, was produced using boranocarbonate supplied by Dr. Abdolreza Yazdani, following previously reported methods (1, 3, 12-14). The process involved mixing a freshly eluted solution of Na^99m^TcO_4_ (20 - 140 mCi) in 1 mL of saline with Na_2_CO_3_ (15 mg), Na-K tartrate (22 mg), Na_2_H_3_BCO_2_ (10 mg), and NaBH_4_ (20 mg) in a nitrogen-purged vial. The mixture was heated for 30 minutes at 100°C and allowed to cool to room temperature. The pH of the solution was adjusted to 7 using HCl (3).

The produced complex was evaluated for RCP using thin-layer chromatography (TLC) and high-performance liquid chromatography (HPLC). Thin-layer chromatography was conducted on Silica gel 60 F254 pre-coated aluminum sheets (Merck) with two mobile phases: 0.1 M citrate buffer (pH 6) and a 1% HCl solution in methanol. The formation of ^99m^Tc-tricarbonyl was confirmed by adjusting the solution's pH to 7 and analyzing with both TLC and HPLC. Radioactivity distribution for TLC was determined using a cut and count method. Radioactivity levels were measured using a NaI well counter (Triathler Multilabel Tester; Hidex; Finland) and a dose calibrator (Atomlab 100; Biodex; USA).

In the HPLC mobile phase (series 1200, Agilent, Japan), a gradient of acetonitrile and 0.1% trifluoroacetic acid was applied for the first 2 minutes. After 18 minutes, the acetonitrile concentration in 0.1% trifluoroacetic acid was 2%. The acetonitrile concentration then gradually increased to 100% over the next 20 minutes, followed by a 2-minute wash with 100% acetonitrile. In the final 5 minutes, acetonitrile was gradually reduced to 2% in 0.1% trifluoroacetic acid. This was passed through a C18 column (stationary phase) (MZ-Analysentechnik GmbH, Germany) at a flow rate of 1 mL/min (1).

### 3.3. Radiolabeling of His-tagged Mutant Nanobody with 99mTc-Tricarbonyl Complex

Briefly, 500 µL of ^99m^Tc-tricarbonyl solution with an activity of 10 mCi was added to a vial containing 100 µg of mutant nanobodies in phosphate-buffered saline (PBS). To monitor the labeling progress of nanobodies, TLC was performed in a 0.1 M citrate buffer (pH 6) at intervals of 30, 60, 90, and 120 minutes. At each specified time, a sample was taken from the reaction mixture.

Purification of the labeled nanobodies was carried out using a PD-10 column (Sephadex G-25, GE Healthcare). The labeled nanobody was added to the column, which was then washed with PBS. Fractions of 1 mL were collected, and their activity was measured using a curiemeter. Radiochemical purity was determined by TLC using TLC-SG with a citrate buffer mobile phase.

### 3.4. Stability Studies of Radiolabeled Nb-mut2

The purified radiolabeled Nb-mut2 solution (100 µL) was mixed with 100 µL of PBS and stored at laboratory temperature for 4 hours. Thin-layer chromatography analysis was conducted every two hours using a 0.1 M sodium citrate mobile phase (pH 6). Additionally, a 100 µL sample of the labeled nanobody solution was mixed with 100 µL of 0.1 M histidine solution and incubated at 37°C for 4 hours. Thin-layer chromatography was performed on this mixture at 2-hour intervals using a 0.1 M sodium citrate mobile phase (pH 6).

## 4. Results

### 4.1. Expression and Purification of Mutant Nanobody

**Figure 1.**
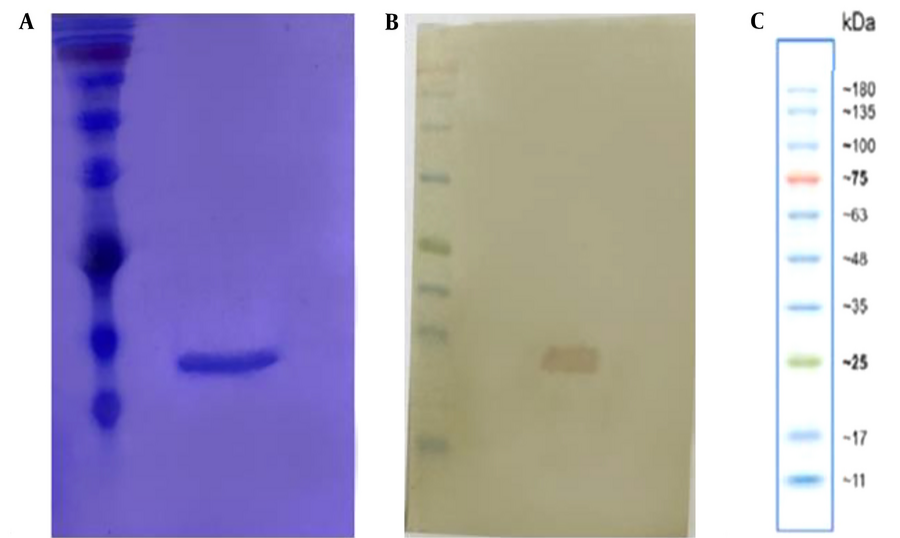
Expression and purification of the mutant nanobody. A, SDS-PAGE of the mutant nanobody; B, Western blot analysis of nanobodies; C, prestained protein ladder.

The purified protein exhibited a single band at approximately 15 kDa on SDS-PAGE (Figure 1A). Western blotting confirmed the size and purity of the mutant nanobodies (Figure 1B). 

### 4.2. Quality Control

**Figure 2.**
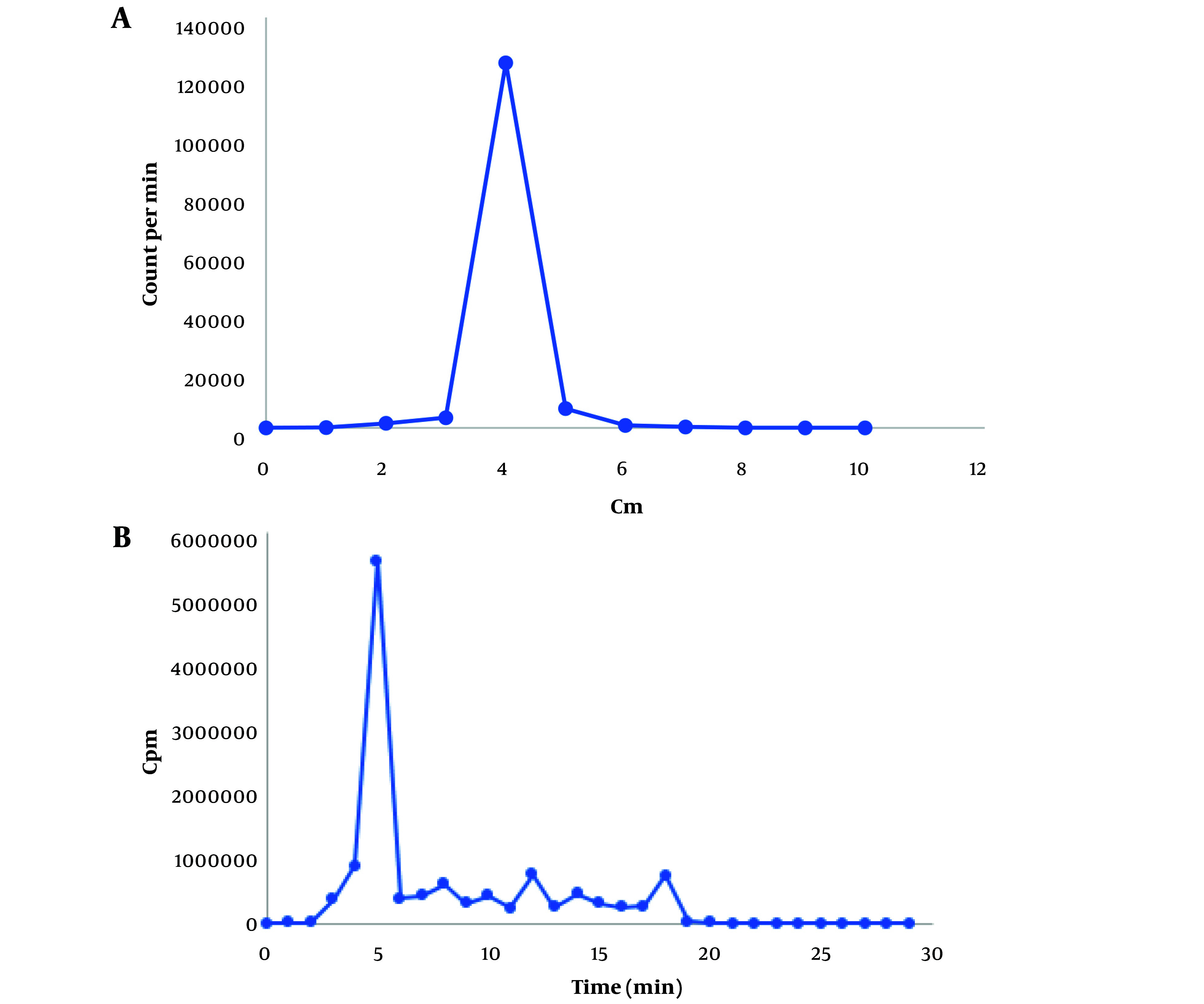
A, thin-layer chromatography (TLC) radioactivity profile of ^99m^-technetium tricarbonyl stationary phase TLC-SG, mobile phase citrate buffer 0.1 M with pH 6; B, radio-high-performance liquid chromatography (HPLC) chromatogram of ^99m^Tc-tricarbonyl (retention time 5 min), C18 stationary phase, mobile phase acetonitrile gradient, and 0.1% trifluoroacetic acid at a flow rate of 1 mL/min.

In thin TLC, sodium pertechnetate (Na^99m^TcO_4_) migrates with the solvent front (R_f_ = 0.9 - 1). In citrate buffer, ^99m^Tc-tricarbonyl displays an R_f_ value of 0.4, and in 1% hydrochloric acid solution in methanol, it appears at R_f_ = 0.2. If ^99m^TcO_2_ (reduced and hydrolyzed technetium) forms, it remains at the origin (R_f_ = 0). Consequently, the radiochemical purity of ^99m^Tc-tricarbonyl was determined to be 99% in citrate buffer and 98% in HCl in methanol (Figure 2A). 

In HPLC, ^99m^Tc-tricarbonyl appeared at the fifth minute with a purity of 98%, aligning with the TLC results (Figure 2B). 

### 4.3. Confirmation of Nanobody labeling by 99mTc-Tricarbonyl and Its Purification

**Figure 3.**
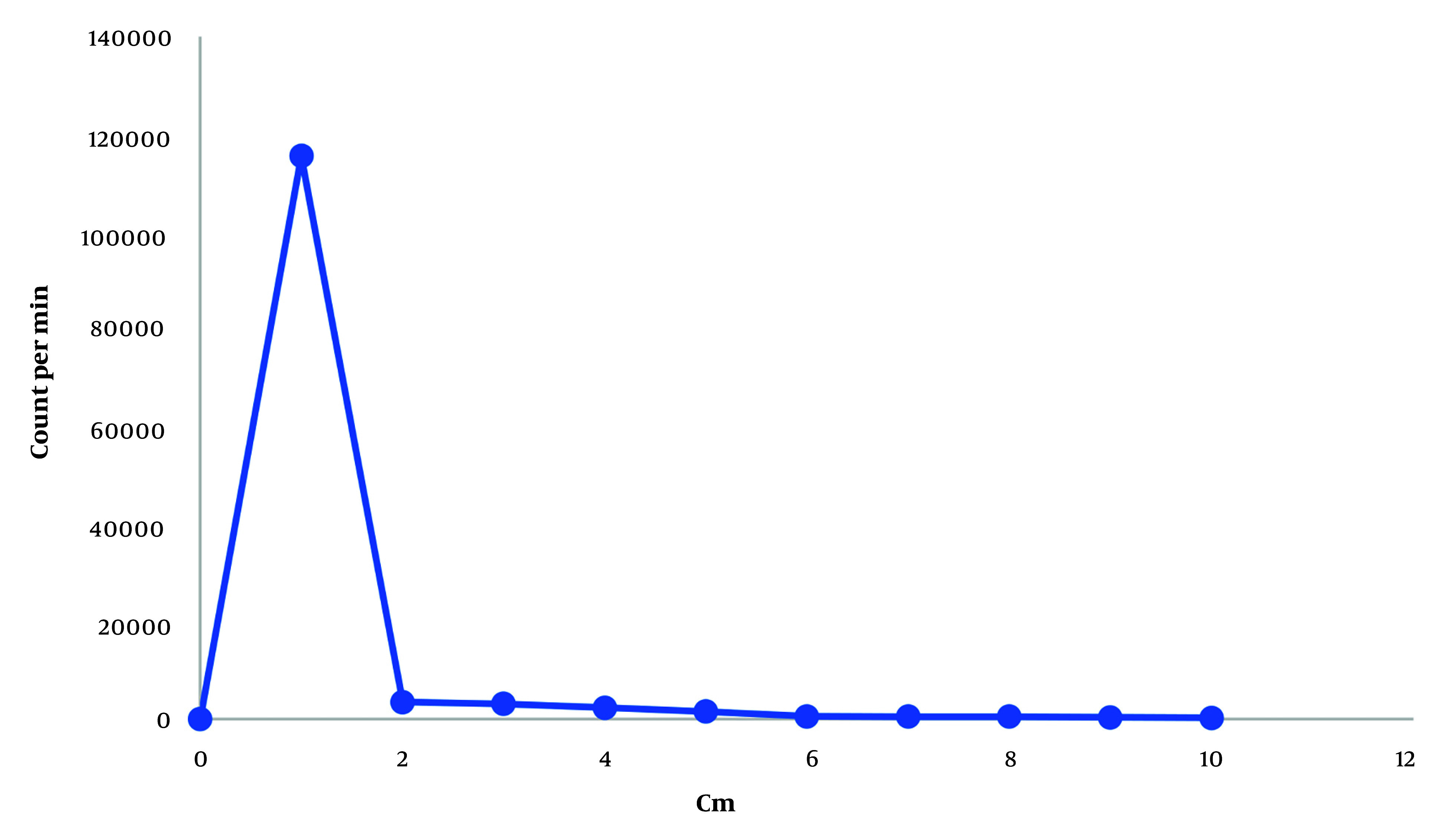
Thin-layer chromatography (TLC) radioactivity profile of the purified radiolabeled nanobodies.

In TLC, the radiochemical purity increased over time, reaching 70% after two hours and then stabilizing (nanobody remains at the origin (R_f_ = 0). During the purification process, the highest amount of radiolabeled nanobodies was collected in the second fraction from the PD-10 column. TLC analysis showed the radiochemical purity of the first and second fractions to be 100% (Figure 3). 

### 4.4. Binding Stability of Mutant Nanobody to 99mTc-Tricarbonyl

**Figure 4.**
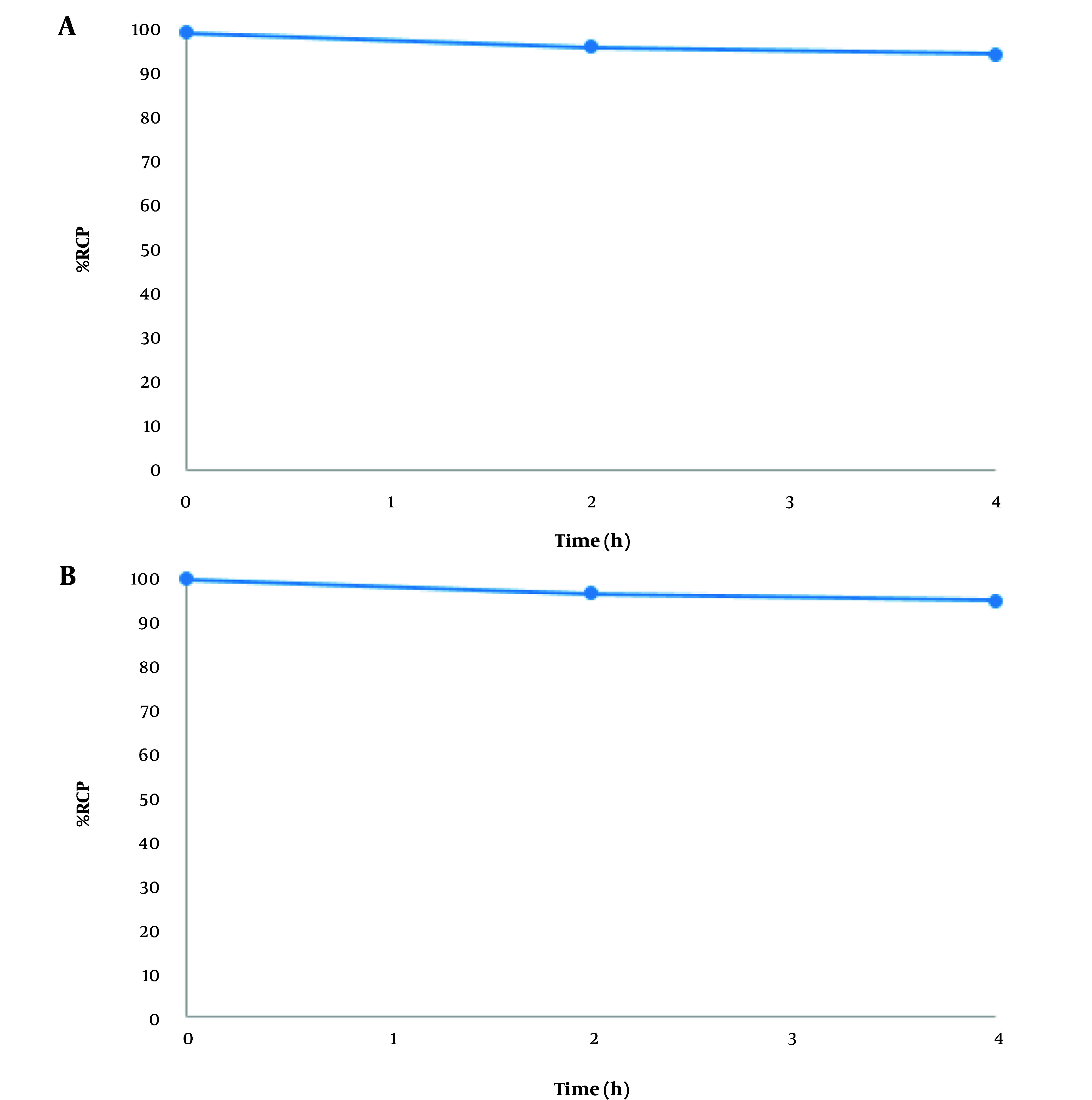
A, stability studies of purified radiolabeled nanobodies incubated with phosphate-buffered saline (PBS); B, stability studies of purified radiolabeled nanobodies incubated with histidine.

The radiochemical purity of the labeled nanobody remained above 95% after 4 hours in PBS (Figure 4A) and histidine solution (Figure 4B), demonstrating the stability of the labeled antibody.

## 5. Discussion

Improving societal health remains a primary objective globally, especially in the fight against non-communicable diseases such as cancer, which is a leading cause of mortality in developing countries. Therefore, early detection is crucial for effective cancer treatment and management. PET and SPECT are molecular imaging methods that utilize tracers composed of a detector and a radioisotope component.

Nanobodies are particularly effective as detectors due to their high tissue penetration, rapid renal clearance, small size, and brief half-life, avoiding some of the challenges associated with full antibodies. Technetium-99m stands out as an optimal radioisotope for labeling because of its suitable half-life and robust signaling capabilities.

Currently, some nanobodies are undergoing clinical trials for cancer imaging, and others have received FDA approval. The sdAb 2Rs15d, targeting HER2, has shown promising results in clinical settings. A study by Keyaerts et al. (15) using ^68^Ga-NOTA-2Rs15d reported favorable biodistribution and swift bloodstream clearance. Ongoing phase II clinical trials (NCT03331601 and NCT03924466) are investigating its efficacy in detecting brain metastases and correlating image-based HER2 quantification with uptake in local or distant metastases in breast cancer patients, respectively (16).

Additionally, two other single-domain antibodies, MM-302 and NM-02, have advanced to phase I clinical studies. ^64^Cu-MM-302 is being tested for its effectiveness against advanced HER2+ cancers with brain metastases (NCT02735798) (17), while ^99m^Tc-labeled NM-02 is under investigation for breast cancer (NCT04040686) (16).

In research by Altunay et al., a specific antibody labeled with ^99m^Tc exhibited extensive distribution throughout the body and significant accumulation at active HER2-positive tumor sites. RAD201 SPECT/CT imaging for a single patient proved safe, with no serious or visible adverse reactions related to tracer administration. The biodistribution data indicated substantial tracer uptake in the kidneys, liver, thyroid, and spleen, with minimal background levels in other organs (18).

Qin et al. demonstrated that [^18^F]AlF-RESCA-MIRC213, a labeled nanobody developed for diagnosing HER2-positive cancers, exhibited high stability both in vitro and in vivo. Notably, there was no significant bone radioactivity in tumor-bearing animals or breast cancer patients, and no adverse reactions were reported during the study. Clinical transformation studies revealed that [^18^F]AlF-RESCA-MIRC213 PET/CT offered favorable pharmacokinetic and dosimetry profiles, making it a promising candidate for noninvasive diagnosis of HER2-positive cancers (19).

Moreover, Liu et al. investigated an anti-EpCAM nanobody for its ability to target EpCAM receptor expression, labeling it with ^99m^Tc. [^99m^Tc]Tc-NB4 demonstrated high specificity for EpCAM both in vitro and in vivo. SPECT/CT imaging showed rapid clearance of [^99m^Tc]Tc-NB4 from the blood and normal organs, except the kidneys. HT-29 tumors were distinctly visualized, contrasting with HL-60 tumors, with the uptake value of [^99m^Tc]Tc-NB4 in HT-29 tumors increasing steadily from 3.77 ± 0.39% ID/g at 0.5 h to 5.53 ± 0.82% ID/g at 12 h post-injection. Additionally, [^99m^Tc]Tc-NB4 SPECT/CT effectively imaged tumor-draining lymph nodes (20).

Furthermore, Joukar et al. (1) optimized the radiolabeling of scFv with ^99m^Tc-tricarbonyl. Their research indicated that the labeled scFv maintained stable binding with ^99m^Tc-tricarbonyl using in-house produced boranocarbonates. Optimal radiolabeling conditions included a scFv concentration > 2 mg/mL, PBS (21), 2 h incubation at 50°C, pH 8 - 9, and a high activity concentration of tricarbonyl. The radiochemical purity of scFv was 70% before purification (1).

Arezumand et al. identified a high-quality nanobody against PlGF from an immune library via phage-display (22). After enhancing the affinity of this nanobody through in silico experiments, two engineered nanobodies (Mut2:S31D and Mut4:R45E) were developed, showing promise based on bioinformatic parameters and molecular dynamics (MD) simulation (23, 24). These mutant nanobodies were then tested for their effectiveness in inhibiting angiogenesis by measuring human umbilical vein endothelial cell proliferation and 3D capillary tube formation (9).

In this study, in response to the need for more efficient methods for cancer diagnosis and treatment, the anti-PlGF nanobody was expressed in *E. coli TG1*. Following periplasmic extraction, the protein was purified using a Ni-NTA affinity chromatography column and confirmed by SDS-PAGE and Western blotting. After removing imidazole via dialysis and concentrating the solution using a freeze dryer, the protein was prepared in PBS buffer.

To minimize impact on the biological activity of the nanobody, the radiolabel was attached to its His-tag tail. ^99m^Tc-tricarbonyl was prepared as a radiolabel, and its RCP was determined by TLC and radio-HPLC methods, showing 99% and 98% respectively. The nanobody was labeled under various conditions with ^99m^Tc-tricarbonyl, and its RCP was measured by the TLC method, achieving 100% purity post-purification. The stability of the purified labeled nanobody was assessed in incubation with histidine and in PBS buffer; after 4 hours, RCP was approximately 95%. Further studies are needed to fully understand how this labeling method affects the physiological performance of the nanobody.

### 5.1. Conclusions

Nanobodies with a His-tag exhibit superior pharmacokinetic properties compared to whole antibodies, making them promising candidates for targeted imaging. ^99m^Tc-tricarbonyl provides a rapid and straightforward method for radiolabeling nanobodies without compromising their biological activity. In this study, His-tagged mutant anti-PlGF Nb was radiolabeled with ^99m^Tc-tricarbonyl using boranocarbonate, demonstrating stable binding between the nanobody and ^99m^Tc. Thin-layer chromatography showed that the radiochemical purity percentage reached 70% after two hours and remained stable throughout the purification process, with the highest amount of labeled nanobodies recovered in the second fraction of the PD-10 column. The radiochemical purity of fractions one and two was 100%, and the labeled nanobody maintained over 95% purity after 4 hours in PBS and histidine solution, indicating stability. These findings are promising and may facilitate future in vitro and in vivo studies in cellular and animal models.

## Data Availability

The dataset presented in the study is available on request from the corresponding author during submission or after publication.
